# Dataset of chlorophyll content estimation of *Coffea Canephora* using Red and Near-Infrared consumer-grade camera

**DOI:** 10.1016/j.dib.2018.10.035

**Published:** 2018-10-16

**Authors:** Bayu Taruna Widjaja Putra, Peeyush Soni

**Affiliations:** a Faculty of Agricultural Technology, Jember University, Jember 68121, Indonesia; b Department of Agricultural and Food Engineering, Indian Institute of Technology Kharagpur, Kharagpur 721302, India

## Abstract

This dataset presents a series of broad Red and Near-Infrared (NIR) bands obtained from consumer-grade camera in estimating chlorophyll contents in Robusta coffee (*Coffea Canephora*) plants at the leaf level. A total of 600 leaves were measured using direct-leaf tools namely SPAD-502 chlorophyll meter, Spectrometer, and modified consumer-grade camera using a 665 nm long-pass external filter.

**Specifications table**

**Table:** 

Subject area	*Biophysical properties, direct-leaf measurement, plant health monitoring*
More specific subject area	*Plant sciences; chlorophyll content estimation*
Type of data	*Tables and figures*
How data was acquired	*Images were captured using a set of mini chamber with a modified consumer-grade camera and 665 nm long-pass external filter, Spectrometer with the range of 350–1000 nm, SPAD-502 chlorophyll meter.*
Data format	*Excel format and analyzed*
Experimental factors	*Plants grown in irrigated and rain fed plantations*
	*No symptoms of pests and diseases*
	*Robusta coffee plant age between 2 to 10 years*
	*Choosing the second or the third leaf from the apex at each branch*
Experimental features	
Data source location	*Indonesia Coffee and Cocoa Research Institute (ICCRI), Jember, Indonesia*
Data accessibility	*The data is available with this article*
Related research article	*B.T. Widjaja Putra, P. Soni, Evaluating NIR-Red and NIR-Red edge external filters with digital cameras for assessing vegetation indices under different illumination, Infrared Phys. Technol. 81 (2017) 148–156.*doi:10.1016/j.infrared.2017.01.007.
	*B.T. Widjaja Putra, P. Soni, Enhanced broadband greenness in assessing chlorophyll a and b, Carotenoid, and Nitrogen in Robusta coffee plantations using a digital camera, Precis. Agric. (2017).*doi:10.1007/s11119-017-9513-x.

**Value of the data**

• The data show red, green, and blue bands (RGB), and several indices (obtained using spectrometer) values. Therefore, they allow the exploration to develop suitable indices for assessing chlorophyll content.
• The data show equation models and relationship between SPAD and vegetation indices obtained using spectrometer and modified camera with a 665 nm long-pass filter. Therefore, they allow extending the statistical analyses.
• Data were obtained from direct-leaf measurement, thus allowing the comparison with different measurement levels, like above-canopy and aerial measurements.
• Data were collected using reflectance of halogen and custom chamber. Thus, the researchers can compare different measurement methods, one of which is absorbance method.

## 1 Data

**Fig. 1:**
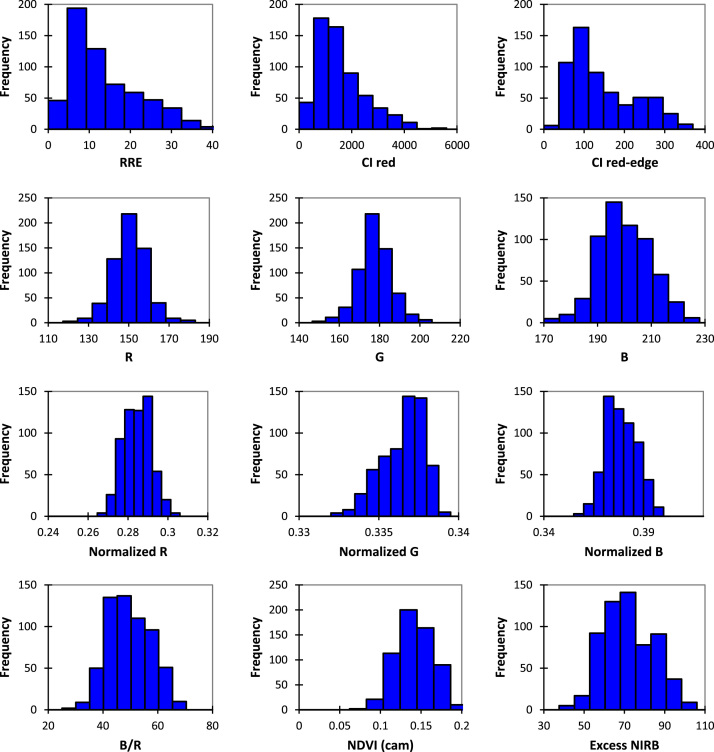
Histogram of vegetation indices obtained using spectrometer and modified camera with a 665 nm long-pass filter.

Data presented in this article are related to the estimation of chlorophyll content in Robusta coffee plants using broadband red and NIR consumer camera. SPAD-502 chlorophyll meter [1] and several vegetation indices such as Simple ratio and chlorophyll indices (CI) obtained from spectrometer were also used as scientific tools to show the potential of modified camera with custom chamber for monitoring leaf greenness and chlorophyll content in Robusta coffee plant. Each of R, G, and B bands was obtained using camera with a 665 nm long-pass filter represented Red, Red and NIR, and NIR respectively [2]. The mean of each Red (R), Green (G), and Blue (B) digital number (DN) was extracted from a set of 600 leaf images for further use. The visualization of each collected and extracted data from the images and scientific tools are presented in Fig. 1.

## 2 Experimental design, materials, and methods

### 2.1 Experimental design

A consumer-grade digital camera (Canon IXUS 160) was modified for obtaining red and NIR spectra by removing ultraviolet (UV) and IR internal rejection filter and adding a SCHOTT 665 nm long-pass filter. In addition, a set of small chamber with halogen light (50 W) was used along with the modified camera [3]. Since halogen lamp was used as light source in custom chamber, white balance mode was used once before data collection.

SPAD 502 chlorophyll meter and Spectrometer as scientific tools were used along with the modified camera. SPAD values were obtained by measuring absorbance leaf in red and NIR regions, while Spectrometer values were obtained by measuring leaf reflectance.

### 2.2 Vegetation indices

Vegetation indices comprise of up to three bands available in consumer-grade camera. Common vegetation indices (VIs) such as normalized difference vegetation index (NDVI) [4], excess NIR [2], blue band minus red band (BMR) or simple ratio [3], normalized band [2] have been successfully used in many studies which incorporate infrared and visible band captured using camera. Several vegetation indices used along with this data are shown in Table 1.

**Table 1:** Vegetation indices used along with data.

No.	Vegetation indices	Formula	References
1	Normalized difference vegetation index (NDVI_Cam_)	(B − R)/(B + R)	[4]
2	excess NIR	2B − G − R	[2]
3	Blue minus red (BMR) or simple ratio blue and red	B − R or B/R	[3]
4	Normalized red	R/(R + G + B)	
5	Normalized green	G/(R + G + B)	
6	Normalized blue	B/(R + G + B)	
7	Ratio red-edge (RRE)	RE_750_/RE_710_	[6]
8	CI index _Red-edge_	NIR_815_/RE_715_ − 1	[7]
9	CI index _Red_	NIR_815_/RE_670_ − 1	

### 2.3 Data collection

A leaf or a pair of leaves chosen from the second or the third leaf from the apex were selected from Robusta coffee plant with age ranging from 2 to 10 years under different grown conditions (irrigated and rain fed plantation), These selected leaves did not indicate any multiple nutrient deficiencies and/or pest and disease symptoms.

**Fig. 2:**
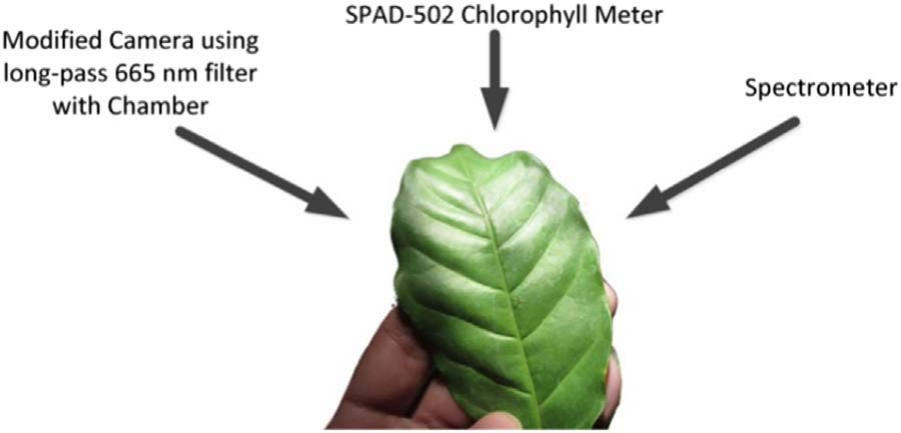
Each leaf of Robusta coffee measured using three different tools.

A set of modified camera with chamber, SPAD, and Spectrometer was used for direct-leaf measurement of 600 leaves of Robusta coffee plants (Fig. 2). Each leaf was measured using modified camera, and the measurement results were then averaged. The leaves were measured by scanning them 10 times and 8 times to gain Spectrometer and SPAD values, respectively [3], [5].

### 2.4 Analysis

**Fig. 3:**
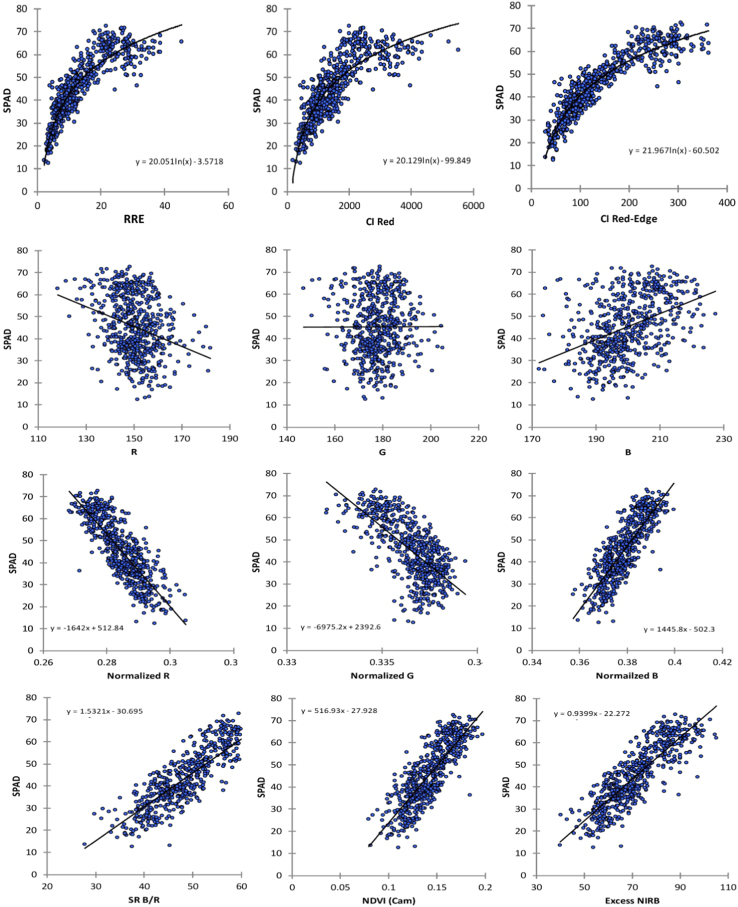
Scatter plot between SPAD and vegetation indices obtained using spectrometer and modified camera with a 665 nm long-pass filter.

RGB digital number (DN) of each image was extracted using ImageJ software (National Institute of Health, USA). The mean of RGB values were used for determining VIs and then compared with SPAD and VIs (SR_Red-Edge_ and CIs) values using regression analysis for estimating chlorophyll contents (Fig. 3).
